# Supplementary figures and images for: Growth-promoting characteristics of potential nitrogen-fixing bacteria in the root of an invasive plant Ageratina adenophora
Source: PeerJ. 2019 Jun 12;7:e7099. doi: 10.7717/peerj.7099 (PMC6571004; doi:10.7717/peerj.7099)

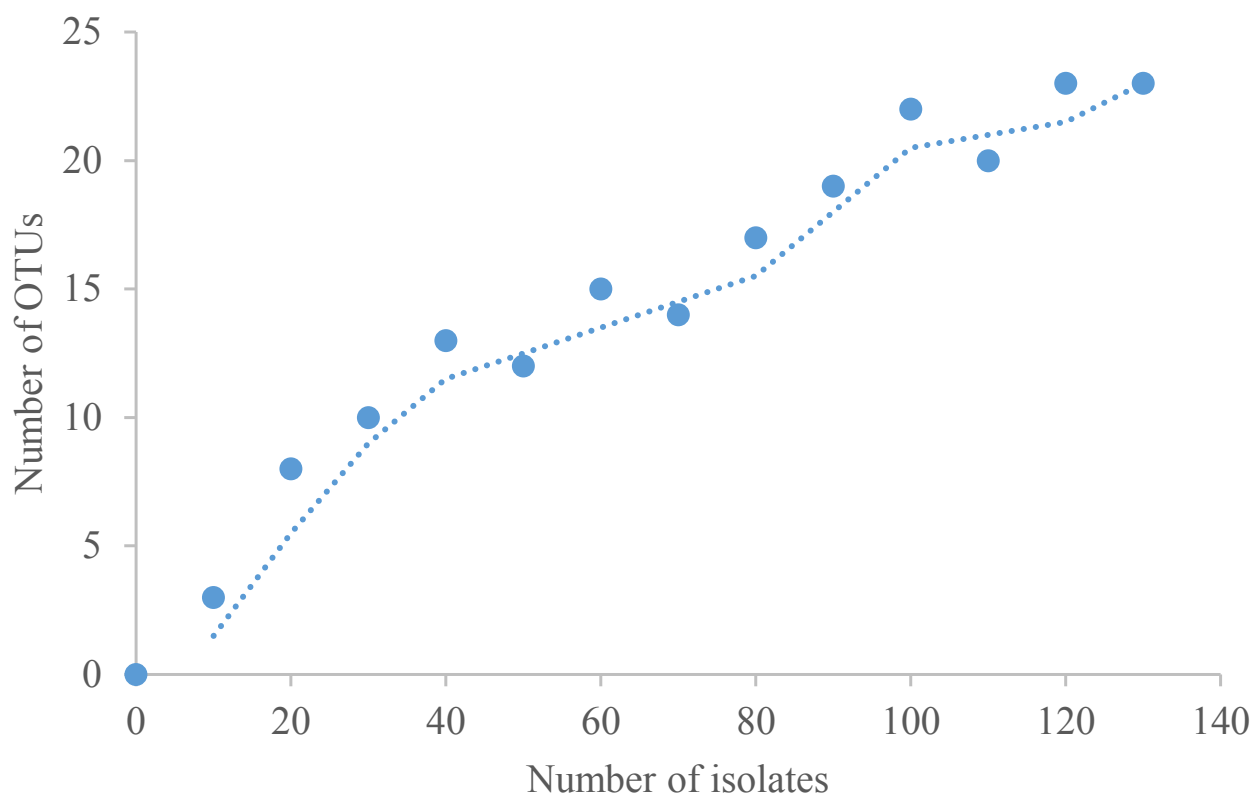

Supplement: Supplemental Information 2 [file peerj-07-7099-s002.pdf]
